# Incubation of human sperm with micelles made from glycerophospholipid mixtures increases sperm motility and resistance to oxidative stress

**DOI:** 10.1371/journal.pone.0197897

**Published:** 2018-06-01

**Authors:** Gonzalo Ferreira, Carlos Costa, Verónica Bassaizteguy, Marcelo Santos, Romina Cardozo, José Montes, Robert Settineri, Garth L. Nicolson

**Affiliations:** 1 Departamento de Biofísica, Laboratorio de Canales Iónicos y Señalización Celular, Facultad de Medicina, Universidad de la República, Montevideo, Uruguay; 2 Fertilab, Montevideo, Uruguay; 3 Sierra Productions Research, LLC, Irvine, California, United States of America; 4 Dept. of Molecular Pathology, The Institute for Molecular Medicine, Huntington Beach, California, United States of America; Universite Clermont Auvergne, FRANCE

## Abstract

Membrane integrity is essential in maintaining sperm viability, signaling, and motility, which are essential for fertilization. Sperm are highly susceptible to oxidative stress, as they are rich in sensitive polyunsaturated fatty acids (PUFA), and are unable to synthesize and repair many essential membrane constituents. Because of this, sperm cellular membranes are important targets of this process. Membrane Lipid Replacement (MLR) with glycerophospholipid mixtures (GPL) has been shown to ameliorate oxidative stress in cells, restore their cellular membranes, and prevent loss of function. Therefore, we tested the effects of MLR on sperm by tracking and monitoring GPL incorporation into their membrane systems and studying their effects on sperm motility and viability under different experimental conditions. Incubation of sperm with mixtures of exogenous, unoxidized GPL results in their incorporation into sperm membranes, as shown by the use of fluorescent dyes attached to GPL. The percent overall (total) sperm motility was increased from 52±2.5% to 68±1.34% after adding GPL to the incubation media, and overall sperm motility was recovered from 7±2% after H_2_O_2_ treatment to 58±2.5%)(n = 8, p<0.01) by the incorporation of GPL into sperm membranes. When sperm were exposed to H_2_O_2_, the mitochondrial inner membrane potential (MIMP), monitored using the MIMP tracker dye JC-1 in flow cytometry, diminished, whereas the addition of GPL prevented the decrease in MIMP. Confocal microscopy with Rhodamine-123 and JC-1 confirmed the mitochondrial localization of the dyes. We conclude that incubation of human sperm with glycerolphospholipids into the membranes of sperm improves sperm viability, motility, and resistance to oxidizing agents like H_2_O_2._ This suggests that human sperm might be useful to test innovative new treatments like MLR, since such treatments could improve fertility when it is adversely affected by increased oxidative stress.

## Introduction

Sperm are highly specialized and must traverse a changing environment to fertilize oocytes. From the total number of sperm found initially in an ejaculate (200–600 million), only 200–300 will come close to eggs, and from those, only one sperm will eventually fertilize an egg [1]. Sperm are surrounded by a unique plasma membrane, and inside there are a number of other membranes that separate various sperm organelles, including mitochondria. There is increasing evidence that the membranes of sperm (SM) are more than just an inert barrier system [2]. Lipids in the SM, especially the glycerolphospholipids (GPL), are compositionally disparate in different membrane regions and undergo changes during sperm maturation where they also function as an energy storage system. In sperm, GPL and sphingomyelin are characterized by the presence of long-chain and very-long-chain polyunsaturated fatty acids (PUFA) (indeed, almost 30% of the fatty acids are PUFA) [3–7] [8–11].

During normal sperm maturation, the continuity between the plasma membrane and intracellular organelle membranes change [12, 13], and there are several modifications in membrane lipid compositions that influence membrane-membrane interactions, fluidity and mobility of lipids within the SM [13, 14]. These changes in lipid compositions in SM are essential for membrane fusion and are required for the bending and fusion of the membrane domains in the plasma membrane and organelles, while recruiting proteins from various membranes and the cytosol. The SM also exert appropriate control over signal processing mediators, such as calcium ions (Ca^2+^) [15]. Lipids from different SM are subject to exchange and renewal by various mechanisms, such as endocytosis, exocytosis, contacts between different membranes and non-membrane lipid vesicles and by non-vesicular lipid carrier trafficking [15, 16]. For example, the compositions of lipids in the SM have been shown to be susceptible to compositional changes by the incorporation/fusion of nanoliposomes [17].

As in other cells, the SM are particularly sensitive to oxidative stress, especially from Reactive Oxygen and Nitrogen Species (ROS/RNS). The production of ROS/RNS in sperm has been known since the 1940s and was found to be important in sperm function and viability [18–21]. In addition, lipid peroxidation was found to affect SM integrity, leading to the loss of cytosolic components and finally cell death [22]. The high concentrations of PUFA compared to saturated lipids in SM makes them highly susceptible to lipid peroxidation. Indeed, reductions in sperm motility have been associated with lipid peroxidation of SM [2, 3, 23–32], and the loss of sperm motility with time has been used as an indirect estimation of oxidative stress and rate of lipid peroxidation [33]. Reductions in sperm motility caused by SM lipid peroxidation were also reported to decrease fertility [8, 34–36]. The natural defense mechanisms of sperm against lipid peroxidation are mostly superoxide dismutase (SOD) and the glutathione peroxidase/reductase system (GPX/GRD). Both are essential, but the SOD defense system seems to be more variable between human sperm samples [3, 37]. The GPX/GRD system appears to be limited by the glucose-6-phosphate dehydrogenase-catalyzed rate of production of NADPH. In addition, seminal plasma can also act as a natural scavenger against lipid peroxidation [3]. These natural defense systems can be overloaded in pathological conditions, leading to deficiencies in male reproduction [23, 37–42].

Oxidative stress and the generation of ROS/RNS also have an impact on sperm mitochondrial function. Oxidative stress and excess ROS/RNS increase the activity of mitochondrial proteins, such as BCL-2, which stimulate the release of mitochondrial proapoptotic factors into the cytosol, activating caspases and harming mitochondrial membrane integrity with loss of the mitochondrial inner membrane chemical/electrical potential (MIMP) [43–45]. The over-production of ROS/RNS also reduces the ATP levels in sperm [46]. There is a direct relationship between loss of MIMP and sperm viability, because the loss of MIMP leads to the reduction of ATP production during cell respiration, leading eventually to cell death [45, 46]. Thus when mitochondria are affected by oxidative stress, there is a loss in MIMP, and this is followed by alterations in many sperm functions, such as motility, viability and the ability to undertake fertilization [47].

The SM is especially damaged during cryopreservation procedures, altering the viability of sperm recovered from frozen samples [48]. That is the reason why most studies on membrane replacement have been limited to its possible use in cryopreservation. Previous studies attempting to restore membrane function in sperm have been done with phospholipids in cryopreserved ram sperm [49], or omega-3 PUFA to cryopreserve turkey sperm [50]. A similar approach has been recently tested for cryopreserved-human sperm using phosphatidylserine present in a freezing supplement (Trolox) [51]. Finally, there are reports of freezing supplements that have used phosphatidylcholine from soybean to increase the cryotolerance of human sperm [52]. Although these studies were relatively successful, they did not explore the use of membrane lipid replacement (MLR) at the molecular level, nor they did not use GPL mixtures or mixtures mimicking most of the SM components, and they did not test oxidative stress damage to sperm incubated at 37°C.

We have used dietary MLR to replace oxidized mitochondrial membrane lipids to improve MIMP and restore function [53, 54]. MLR utilizes mixtures of cell membrane GPL, plus fructooligosaccharides for protection against oxidative, acid and enzymatic damage, in order to safely replace damaged, oxidized, membrane GPL through diet. In preliminary studies MLR with GPL have been shown to improve human sperm motility and viability [55]. Here, we examined the ability of sub-μm-sized micelles prepared from GPL to modify human SM. We also tested human sperm to see if GPL could protect from the loss of motility and viability due to the effects of oxidative stress. Finally, we also examined if the incubation of human sperm with sub-μm-sized micelles prepared from GPL could prevent the loss of MIMP produced by oxidizing agents like hydrogen peroxide (H_2_O_2_). Our results suggest that human sperm can be used to test innovative new treatments like MLR in order to improve male fertility when it is adversely affected by increased oxidative stress.

## Material and methods

### Ethics

The procedure to obtain human sperm was done following ethics procedures approved by the National Ethics Committee at the School of Medicine, Universidad de la República (Res# 070153-001013-14). The donors comprised a random group of 24 adult males with normozoospermia and an average age of 32±1 years. An Informed Consent document was approved by the Ethics Committee and signed by all participants.

### Experimental procedures

#### Obtaining fresh human sperm samples, incubation procedure and preparation of the sub-μm-sized micelles

Human sperm were obtained by ejaculation from young males of reproductive age, (range, 27–39 years). Once obtained, human semen samples were submitted to liquefaction for 30 min, followed by a standard swim-up protocol in accordance with standard procedures, to enrich the preparation of healthy sperm [56]. We excluded sperm samples that yield a viable sperm low count (less than 1 x 10^6^ sperm/ml) and/or more than 60% non-motile sperm cells per field after a one h incubation. After processing the semen samples with a sperm swim-up procedure, the samples were centrifuged at 300 × *g* for 5–10 min and finally resuspended in HAM-F10 medium (GIBCO, Thermofisher cat N° 11550), at an approximate concentration of 20–30 × 10^6^ sperm/ml. To obtain the desired concentrations we measured the diluted initial sperm count from the semen with a Neubauer hematocytometer chamber or a Cell-VU chamber with a grid of standard dimernsions. We counted the sperm in five volumes of the 0.1 mm^3^ (or μl) central counting square. Then we multiplied that result by 10000 to know the concentration per ml, and finally we multiplied that for the dilution factor to know the sperm concentration in the undiluted sample. The original undiluted sample was then diluted to obtain approximate concentrations of 20–30 × 10^6^ sperm/ml. Sperm selected by the swim-up procedure were placed in a sterile incubator at 37°C and 5% CO_2_, with constant mixing a shaker inside the incubator. The selected sperm preparations were incubated under different conditions for average time of 2–3 h. Sperm had to be incubated for up to 2h to reach a steady state due to the kinetics of incorporation of GPL into bilayers, nano-micelles and other vesicles that mimic membrane composition. An almost full substitution of the lipids followed hyperbolic Michaelis-Menten-like kinetics [57]. The incubation procedure offered several advantages as it allowed; (a) an exact time of exposure of the SM to the sub-μm-sized micelles; (b) an adequate monitoring of the viable sperm at different times of incubation; (c) an improvement of the impact of the motile sperm with the sub-μm-micelles; and finally (d) testing in parallel of different experimental conditions, such as incubation with oxidizing agents. To promote oxidative stress incubation was performed with 300 μM H_2_O_2_ added to HAM-F10, in accordance with previous studies [58, 59]. To evaluate the antioxidant properties of GPL mixtures, sub-μm-sized micelles were added to sperm incubated in HAM-F10 with 300 μM H_2_O_2_ using a shaker, following the procedure described above. To avoid interference from sub-μm-sized micelles in the measurements of sperm motility all samples were centrifugated after the incubations at 300 x g for 5 min, and the sperm to be tested were taken predominantly from the middle of the centrifugation vial. Centrifugation at low speed does not alter the characteristics of sperm samples [60, 61].

**Fig 1:**
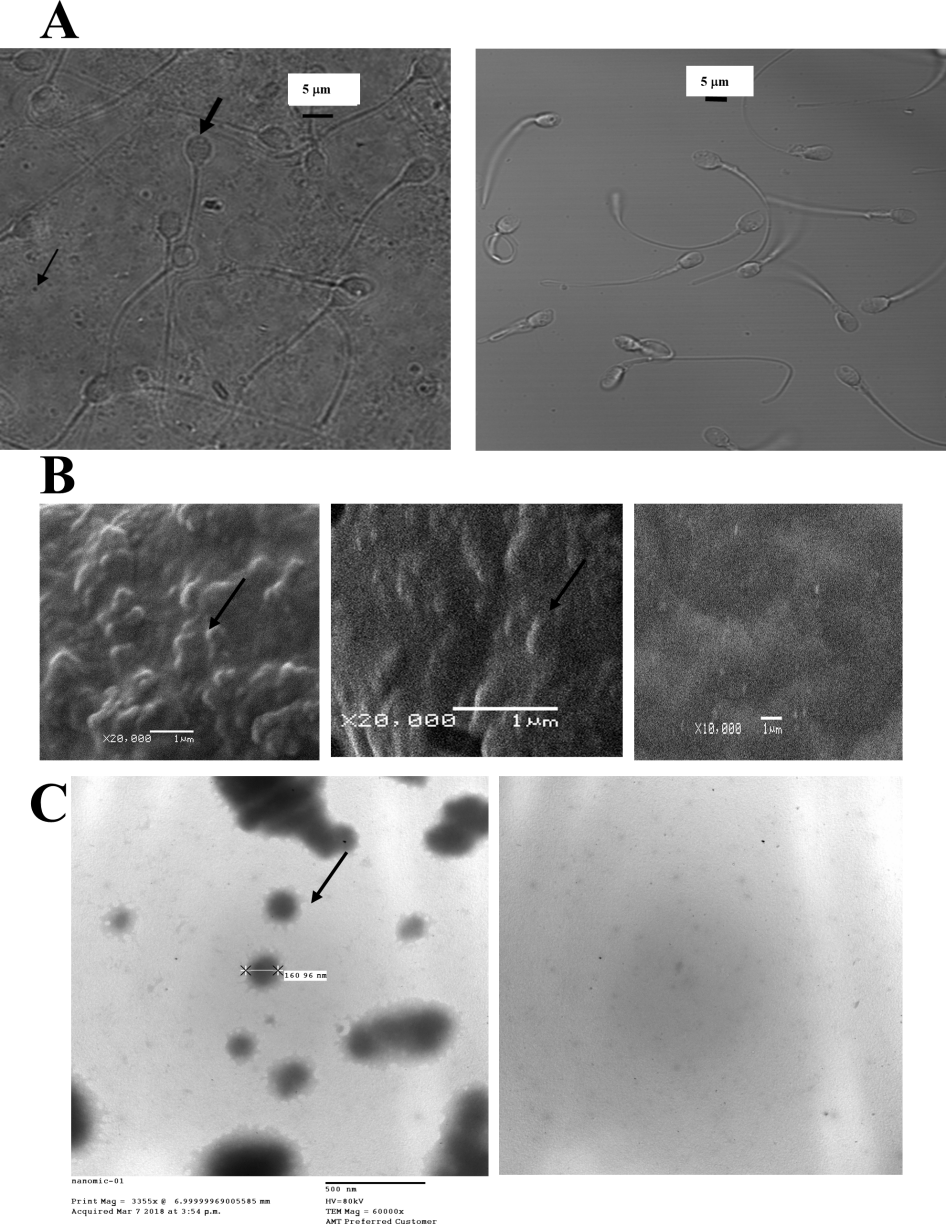
Aggregation of glycerophospholipids into sub-μm-sized micelles. (A) Images of sperm and GPL micelles in a confocal microscope with visible light. Precipitated ultra-sonicated sub-μm-sized GPL micelles (thin arrow) with human sperm (thick arrow) after a 6-h incubation with GPL (left), or human sperm after a similar incubation in media without GPL (right). (B) Precipitated GPL micelles seen in a Scanning Electron Microscope. The left image shows the micelles as protruding images from a thin-layered film (thin arrow). In other regions, isolated micelles can also be seen (middle image, thin arrow). The control image without GPL micelles is shown to the right. (C) GPL micelles observed in a transmission electron microscope with negative staining. To the left single GPL micelles (thin arrow) can be seen, and some micelles colliding and forming larger micelles can be observed. A background image without GPL micelles is shown to the right.

GPL in physiological solutions spontaneously form bilayers or micelles, and if ultrasonicated at low concentrations, tend to form smaller micelles in the diameter range of nm or sub-μm. We used freshly prepared mixtures of GPL and fatty acids of precise composition that mimic the GPL composition of mitochondrial membranes (NTFactor Lipids^®^, Nutritional Therapeutics, Inc. of Hauppuage, NY, USA). This composition of GPL is known (see [62]) and has proved to be successful for in vivo MLR in several human diseases and conditions in various reports [53, 63–66]. The advantage of using a mixture like this with precise proportions of GPL and unsaturated fatty acids is that it closely mimics the compositions of biological membranes. When used in the incubation procedures, the GPL were added to the incubation media with less than 0.1% ethanol to enhance solubility. Control incubation medium was HAM-F10. The addition of ethanol <0.1% did not cause significant variations in data (P = 0.95, n = 8). GPL micelles were prepared at concentrations up to 3% GPL mixtures in the incubation media (typically, 0.1–1% was used). The GPL mixture was ultrasonicated at 20 KHz for 15–25 min, using a probe sonicator plus a 50W Virtis virsonic 475 device (Virtis/SP Industries, Gardiner, NY, 12525, USA), similar to that reported for the constitution of nanocapsules [67–71]. The resulting product was purified as sub-μm-sized micelles with a CL-4B sepharose chromatography size exclusion column, or alternatively using a sterile 0.2 μm Millipore filter similar to filling patch clamp pipettes [72, 73]. Applying this procedure, we obtained sub-μm-sized micelles that mixed well with the media and that were small enough to be incorporated into the SM. Fig 1A (*right*), shows human sperm isolated after an incubation period of 6 h, without the addition of a GPL mixture. The background is clear and homogeneous in this image. Fig 1A (*left*), shows ultrasonicated 3% GPL mixtures after a 6-h incubation with human sperm without shaking to enhance the observation of the sub-μm-sized micelles. The background shows a mesh of precipitated sub-μm-sized micelles, some of them isolated and some fusing with themselves. Following Abbe's law, the average size of the micelles we obtained was in the resolution limit of a light microscope (~210 nm for 100x objective lense with NA 1.45 and visible light wavelength). To obtain a better resolution of the sub-μm-sized micelles, we observed the micelles with a Scanning Electron Microscope (Jeol JSM-5900LV, Tokyo, Japan) and with a transmission electron microscope (Jeol JEM-1010 Tokyo, Japan). To observe the micelles with the scanning electron microscope the micelles were dried and placed into a low-pressure vacuum and sputter coated with gold using a coating device (Denton Vacuum, Desk II, NJ, USA). Fig 1B shows the results obtained on a control surface (right) and on a surface with precipitated sub-μm-sized GPL micelles. Precipitated micelles can be seen isolated (middle) or parts of thin layered films (left). Sub-μm-sized micelles rarely exceeded 1 μm in diameter and were usually at the limit of optical resolution (200–250 nm). The composition of the atomic elements in the micelles in physiological saline solutions (after spraying with gold) was obtained by X-ray Fluorescence (see the blue dots in S1A Fig). The same analysis was also done as a control for the graphite surface sprayed with gold (see S1B Fig). Nitrogen, Oxygen, Sodium, Potassium, and Chloride could only be detected in the dots corresponding to the micelles and not in the control. These results were repeated and observed in a transmission electron microscope. Negative staining of the micelles was done using phosphotungstic acid [74]. The average size of the micelles measured with the transmission electron microscope was consistent with the measurements in the light and scanning electron microscopes. A micelle measuring 160 nm is identified in Fig 1C. They are usually denser in the middle, and a star-shaped outer layer is often observed surrounding them. Small micelles can join into larger micelles as a consequence of the dehydration process necessary to observe them in an electron microscope. The background image without micelles is shown to the right.

#### Preparation of fluorescent sub-μm-sized GPL micelles

To observe the direct incorporation of GPL into SM, we covalently attached the dye Rhodamine 123 to the carboxyl and phosphate residues in the GPL mixture (especially phosphatidylserine, phospatidic acid and fatty acids components, though GPL like phosphatidylcoline can react as well) [75, 76]. The cross-linking conjugation reaction was elicited with water-soluble carbodiimide 1-etil-3-(3-dimetilaminopropil)carbodiimide) (EDC, Thermofisher, USA), under mildly acidic conditions (pH 4–6) [75]. The stability of the active ester was improved using N-hydroxysulfoxuccinimide (sulfo-NHS, Thermo Fisher, USA). All of the conjugation reactions were achieved with EDC carbodiimide, according to previous publications [75, 77]. The amount and isolation of the bioconjugate Rhodamine 123 crosslinked to the GPL mixture was evaluated by the procedure by Nakajima et al. 1995 [78]. The GPL mixture with crosslinked Rhodamine 123 remained fluorescent, and it was then filtered with a 0.2 μm filter. To observe the fluorescence of the conjugates SM were incubated with the cross-linked GPL-Rhodamine 123 and then washed using centrifugation and resuspension. This was used solely to test the incorporation of the sub-μm-sized micelles, prepared using the GPL mixture, into the SM, and it was not used for the functional assays described in this paper.

#### Computer Assisted Sperm Analysis

Computer Assisted Sperm Analysis (CASA) was used to test the motion characteristics of sperm immediately after the incubation procedure was complete. This was performed with a Microptic Sperm Class Analyzer software (SCA) (Microptic, Barcelona, Spain). A volume of 5–15 μl of sperm was used for the incubation procedure to be tested (concentration 20-40x10^6^ sperm/ml measuring). Sperm were loaded into a Cell-VU sperm counting chamber (Millenium Sciences Inc., NY, NY) and placed on slide warmers at 37°C. Sperm samples in the chambers were observed with a Nikon Eclipse E200-LED microscope using a 10X phase objective (Nikon Corporation, Tokyo, Japan), with a Basler ACA 780-75GC camera (Basler AG, Ahrensburg, Germany) connected to a computer with the SCA automated software. The acquisition rate to obtain the videos and idealize the trajectories of sperm in the samples was set to 25 frames/sec. For each Cell-VU chamber loaded, the automatic count for the motility parameters used to evaluate a sperm sample to be tested were set according to WHO standards [79], and the instrument measured a minimum of 8–10 different randomly selected microscopic fields (200–500 sperm/field). The values analyzed were total motility (TM,%), progressive motility (PM,%), velocity according to the smoothed path (VAP, μm/s), velocity according to the straight path (VSL, μm/s), velocity according to the actual path (VCL, μm/s), amplitude of lateral head displacement (ALH, μm), head beat-cross frequency (BCF, Hz), straightness (STR, %) and linearity index (LIN, %). We focused our reports on the most significant variables for our studies, such as overall or total motility, TM, VSL, VCL and VAP for fast, slow and non-progressive sperm with slow, medium or fast velocities. The procedure was repeated three times for each experimental condition during incubation (control, 0.1–1% GPL, 300 μM H_2_O_2_, 300 μM H_2_O_2_ plus 0.1–1% GPL). This type of analysis was performed for samples from 8 different males to get an estimation of the relative variation of dispersion among the different experiments and samples. The variation coefficients obtained varied from 7 to 28% according to the different WHO velocity motility parameters examined and individuals assayed, and this was consistent with several reports [80–82]. Excel spreadsheets were automatically obtained for each sample data collection and also for the total numbers of samples in each different experimental condition.

#### Monitoring the MIMP by flow cytometry using the JC-1 dye

The remaining sperm suspension was used for fluorescence measurements to evaluate the MIMP of the samples using the dye JC-1. In functional mitochondria, a strongly negative MIMP favors the accumulation of the cationic JC-1 probe as an aggregate inside the organelle, yielding red fluorescence. In unhealthy mitochondria, MIMP is less negative and the accumulation of JC-1 into the mitochondria is reduced, favoring its accumulation as a monomer that elicits green fluorescence. A 1000x stock solution of 5,5',6,6' -tetra-chloro-1,1',3,3'-tetraethylben-imidazolyl-carbocyanine iodide (JC-1, Sigma, USA), was prepared at 5 mg/mL in dimethylsulfoxide (DMSO, D8779, Sigma, USA). The JC-1 stock solution was divided into aliquots and stored at -20°C. One ml of the sperm suspension was incubated with JC-1 prepared and diluted 1000 times from the stock solution, yielding a final concentration of 5 μg/ul. Sperm were loaded with the dye under different experimental conditions in the dark in an incubator for 30–40 min at 37°C with 5% CO_2_. After loading, sperm were centrifuged at low speed, and then they were resuspended in various solutions under the different experimental conditions. In each sample, we confirmed staining with JC-1 by removing and observing a small sample on a slide using an epifluorescence microscope, exciting the dye with an Argon laser at 488 nm. The remaining volume of each of the samples was analyzed using a FACSCalibur Flow Cytometer (Becton Dickinson, Mountain View, CA, USA) with the CELLQuest software (Becton Dickinson). We determined the forward scatter (FSC) and side scatter (SSC) of the sperm samples. The FSC and SSC regions corresponding to the JC-1 stained sperm were determined for the acquisition of normal sperm [83]. Samples in the flow cytometer were analyzed to obtain at least 10,000 events for each sample. The samples were excited at 488 nm and emitted light was collected with emission filters at 530 nm (green fluorescence: FL1, dye monomers) and at 585 nm (red fluorescence: FL2, dye aggregates) [84, 85]. The photomultiplier was set for logarithmic scale. The ratio of red/green fluorescence is linearly related to the mitochondrial membrane potential [85]. The larger the red-to-green fluorescent ratio, the more negative the average MIMP, which translates to more active and healthier (functional) mitochondria [84, 85]. Dead/live sperm ratios were determined using 10 μg/ml propidium iodide (PI) from a stock solution of 1mg/ml in water (Sigma Aldrich, USA). PI is a membrane impermeant dye that is generally excluded from viable cells. It binds to double-stranded DNA by intercalating between base pairs. It is excited at 488 nm and emits at a maximum wavelength of 617 nm [86]. A negative control of sperm unstained with the JC-1 dye was also routinely obtained. The results for each experimental condition were analyzed with FlowJo software (FlowJo LLC, Oregon, USA) and displayed either as a dot plot or histograms of events at the green or red wavelength. The GPL mixture used for these experiments was prepared as described above, without covalently attached Rhodamine 123.

#### Confocal imaging of live human sperm

The samples of sperm incubated under different experimental conditions were loaded with JC-1 or Rhodamine 123 dyes (Sigma-Aldrich, USA) to evaluate MIMP [87, 88]. The fluorescence intensity of Rhodamine 123 excited at 488 nm and with an emission at 530 nm is linearly related to the MIMP [87]. The stronger the fluorescent signal at this wavelength, the more negative is the MIMP. We also used JC-1 as a dye to evaluate MIMP as the red/green fluorescence ratio [88]. The loading procedure for both dyes was the same, and it was performed as described for the flow cytometry experiments. After loading, washing, and resuspension of the sperm, they were immobilized for live cell imaging. To immobilize live sperm, the samples were placed for 15 min on thin coverslips covered with poly-L-Lysine (Sigma Aldrich, USA) in semi-sterile conditions in an incubator at 37°C and 5% CO_2_, following the procedure described by Wennemuth et al., 1998 [89]. Coverslips with the samples from each experimental condition were placed in a 35 mm microincubator chamber and held at 37°C for observation under a Leica SP5 confocal microscope (Leica GmbH, Germany). The dye Rhodamine 123 was excited with an Argon Laser at 488 nm, and the emitted light was collected at 530 nm. JC-1 was excited at 488 nm, and the emitted light was collected at both 530 nm (green fluorescence) and at 585 nm (red fluorescence). Images were obtained with either 40X, 63X or 100X oil immersion objectives lenses and were acquired in *xyt* scanning mode at 512x512 pixels. To avoid out-of-focus imaging and collection of light from several planes in the size range of a sperm, the pinhole was usually set at 1.5–2.5 Airy Units. Image processing was done using the Leica LAS AF or LAS X suites (Leica GmbH, Germany) and Image J. The GPL mixture used for these experiments was prepared as described above.

#### Analysis of the results, statistics and figure preparation

The data obtained from applying the methods previously described to each of the eight samples of human sperm under the different experimental conditions was analyzed for statistical analysis using Sigmaplot 11 (Systat Software Inc. USA). Average values and standard error of the mean were obtained for each condition and paired or independent Mann-Whitney Rank t-tests were applied when comparing two samples, in order to know if the changes that occurred with the treatment was greater than would be expected by chance (significance was determined at least with p<0.05). If the comparison was made for more than two treatments within the samples, the statistical analyses to test the significance of the differences of the mean for multiple experimental conditions were performed following the Tukey One-Way ANOVA test, using either Sigmaplot 11 or the SPSS software (IBM, USA). Dose-Response curves were fitted by a Hill model plus an initial constant (Do), R = Do + A*D^n^ /(IC_50_^n^ + D^n^), where R is the response, D is the dose and the parameters are maximum variation, A; half maximal dose, IC_50_ and hill number, n. To get the best Hill parameters that fit the observed values, non-linear regression by non-linear least squares, between the data and the model, was performed. Flow cytometry dot plots and cytograms, were obtained using the CELLQuest (Becton Dickinson) and the FlowJo software (FlowJo, LLC, USA). For image analysis, raw images were imported with Image J (NIH, USA) or the LAS-AF Suite (Leica, Germany) and fluorescence intensity along lines were obtained.

## Results

### Ultrasonication of glycerolphospholipids creates sub-μm-sized micelles that can incorporate GPL into sperm membranes

**Fig 2:**
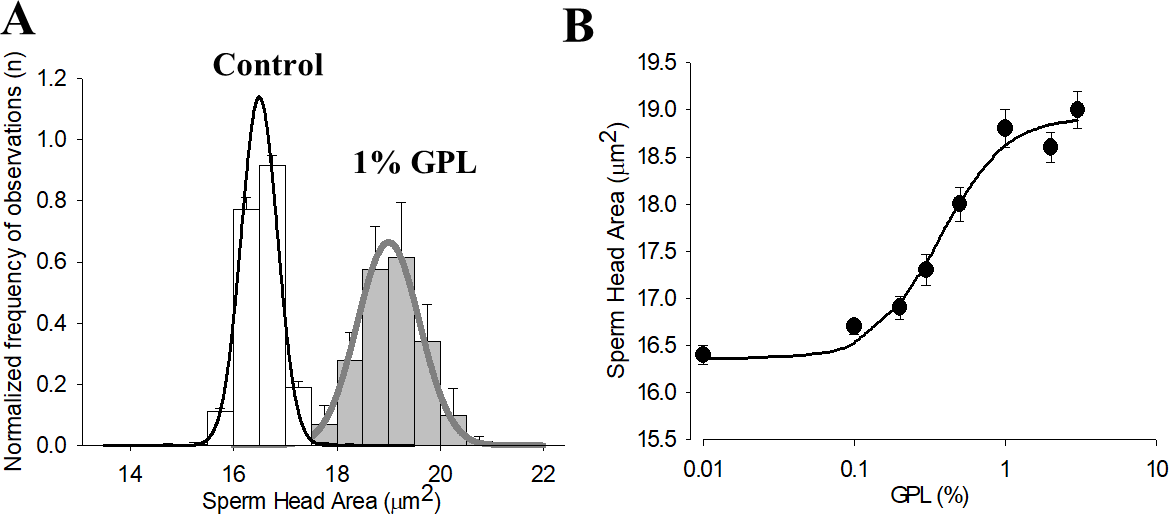
Increase in size of the sperm heads after incubation with sub-μm-sized GPL micelles. (A) Histogram and normal distribution of sperm head size after incubation in control and in 1% GPL mixture. (B) Sperm Head Area and GPL mixture concentration.

When sonicated GPL were mixed with human sperm, the sperm heads increased in size, suggesting the incorporation of the GPL mixture into the sperm plasma membrane. When the average sperm head area in a control solution was compared to sperm head areas in an ultrasonicated 1% GPL solution, the average area of the sperm heads measured by phase contrast during CASA experiments was significantly greater. The size of the sperm heads increased from an average of 16 μm to 19 μm (p<0.05, independent t-test, n = 8). This result held for static or mobile sperm (at all velocities). The GPL were likely incorporated into the plasma membrane and other sperm membranes, but this was not as obvious as with the sperm heads. In Fig 2 the mean areas of the sperm heads versus the concentration of the GPL mixture are shown as normalized histograms (Fig 2A). The size of the sperm head area was dependent on the concentration of GPL (Fig 2B). Increasing the GPL concentrations enlarged the sperm head area, demonstrating that these variables are related.

**Fig 3:**
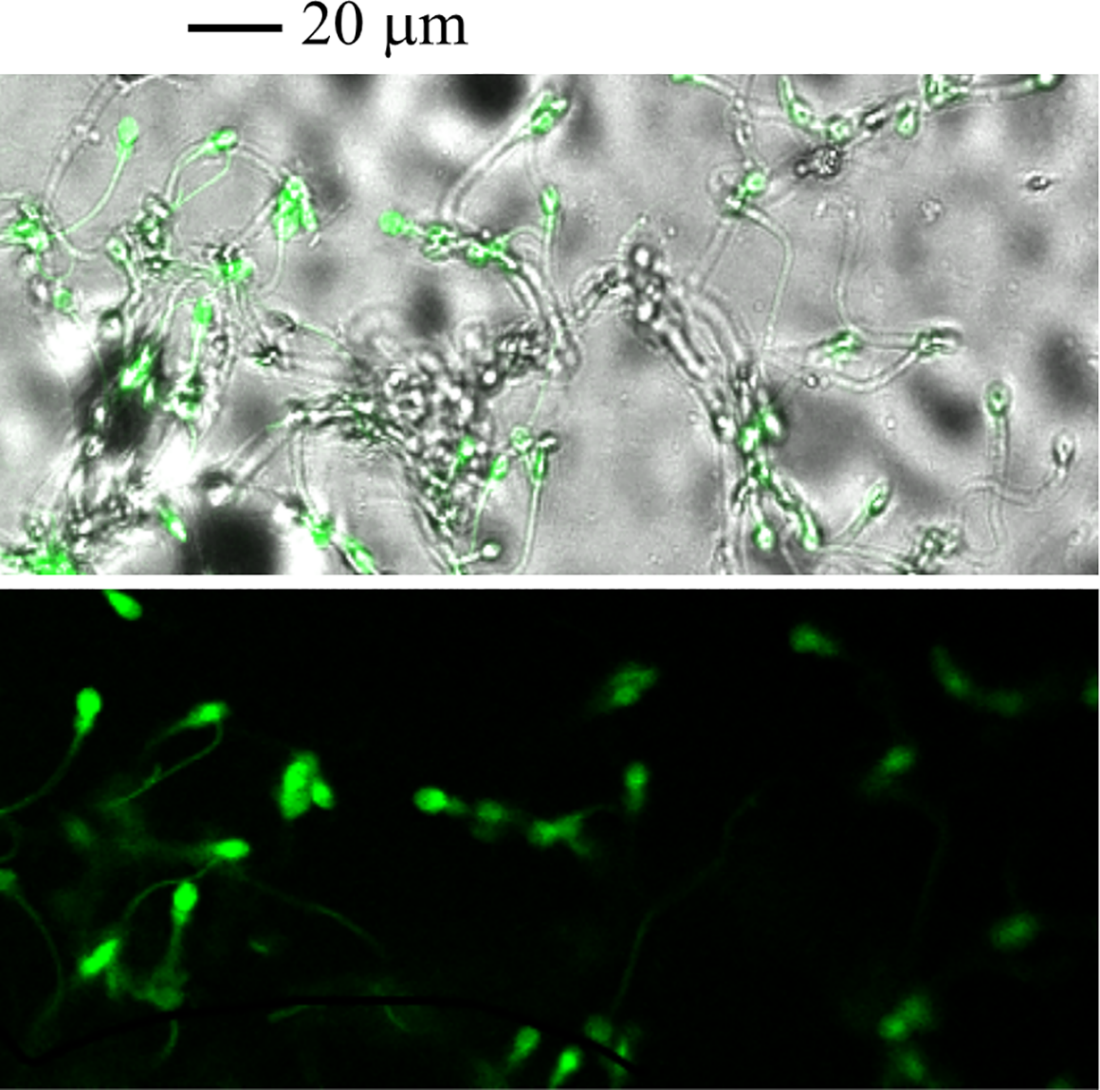
Incorporation of GPL from sub-μm-sized micelles into sperm membranes can be observed with fluorescent GPL crosslinked with Rhodamine 123. Human sperm were incubated with sub-μm-sized micelles made of GPL crosslinked with Rhodamine 123. Images of stained sperm are shown merged with trans-illumination (*upper*) or in fluorescence mode alone (*lower*). Note that more than one-half of the sperm are strongly fluorescent.

Sperm with incorporated, labeled, ultrasonicated GPL are shown in Fig 3. Sperm were incubated with a cross-linked GPL-Rhodamine 123 and then washed using centrifugation and resuspension. High laser intensities at 488 nm had to be used to get light emission from sub-μm-sized micelles. These fluorescent bioconjugates were used solely to observe the incorporation of the GPL into sperm membranes. Most of the fluorescence is seen in the sperm middle piece and in the sub-equatorial region of the sperm head. This suggests that labeled GPL in the mixture can incorporate into these regions of the sperm, at least at the level of the plasma membrane. It has been shown that the mitochondrial membranes and the plasma membrane in the mitochondrial sheet around the mid-piece are interrelated and possibly linked, at least transiently [90].

**Fig 4:**
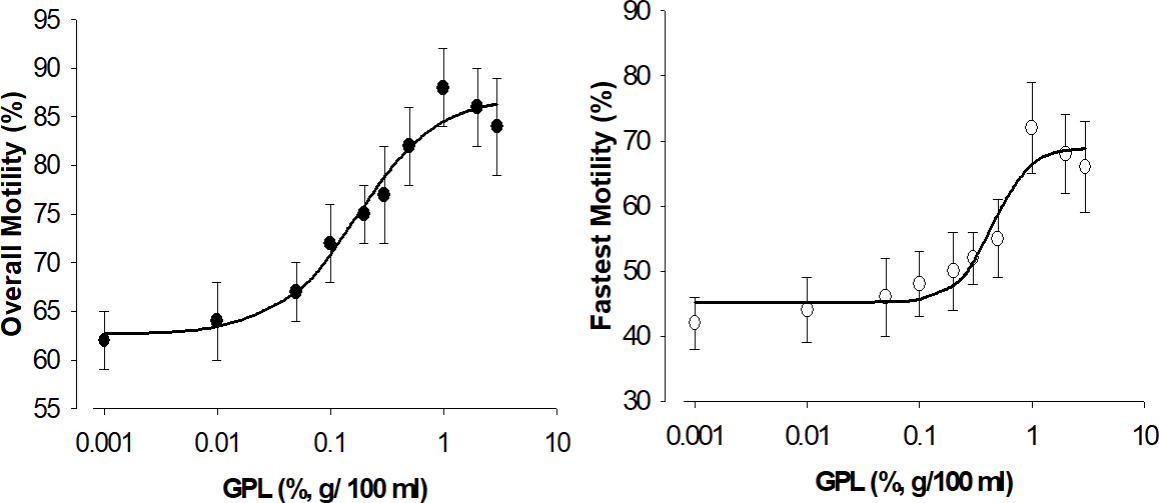
Dose response curves of overall and fastest sperm motility versus concentration of GPL. Both types of velocities are increased by incubation of sperm with sub-μm-sized micelles prepared from GPL. The solid line represents the best fit of a Hill equation. The effect is more pronounced (IC_50_ of approximately 0.5%) for the fastest sperm (*right*) compared with 0.15% for the overall motile sperm (*left*).

Since the lipid composition of the sperm plasma membrane is a major determinant of sperm motility [91], we expected to observe changes in sperm motility with the incorporation of exogenous GPL. To test this hypothesis and get functional data on the incorporation of GPL from the sub-μm-sized micelles into sperm membranes, we measured the motility of human sperm with Computer Assisted Sperm Analysis (CASA) after incubation of various concentrations of sub-μm-sized micelles made with different concentrations of GPL. The results of overall motility and fastest motility versus the concentration of GPL are shown in Fig 4. With increasing concentrations of GPL, both types of motility were increased up to a maximum of 10%. The solid lines represent the best fit of the Hill equation to the average values of overall and fastest motility observed at each GPL concentration. At concentrations above 1% of the GPL mixture there was a tendency to observe a reduction in motility because of the size of GPL micelles interfering with sperm displacement, and possibly because the sperm heads became too heavy to sustain motility. If ultrasonication was not done to the GPL, the micelles also interfered with the normal kinetic movements of the sperm. When sperm were separated from the lipid micelles by low-speed centrifugation, the enhanced motility of sperm cells due to the GPL mixtures was more obvious. After incubation and centrifugation at 300–500 x g for 10 min at 37°C, the increase in sperm motility can be seen in the GPL-incubated samples, especially at concentrations of GPL above 0.5%, supporting the findings reported in Figs 1 and 2. The data indicate that ultrasonicated GPL transfer their lipids to the SM, modifying their composition and function. Next, we evaluated if MLR with the GPL could reduce the damage to SM produced by exposure to oxidizing agents.

#### Incubation of human sperm with an ultrasonicated GPL mixture corrects reduced motility produced after incubation with hydrogen peroxide

Oxidative stress is a major determinant of membrane lipid peroxidation and sperm motility [40–42]. We expect reduced damage from oxidative stress by lipid peroxidation when sperm are incubated with unoxidized GPL like those present in our GPL mixture. We tested this hypothesis by measuring sperm motility under four different experimental incubation conditions (control, GPL, H_2_O_2_, GPL plus H_2_O_2_).

**Fig 5:**
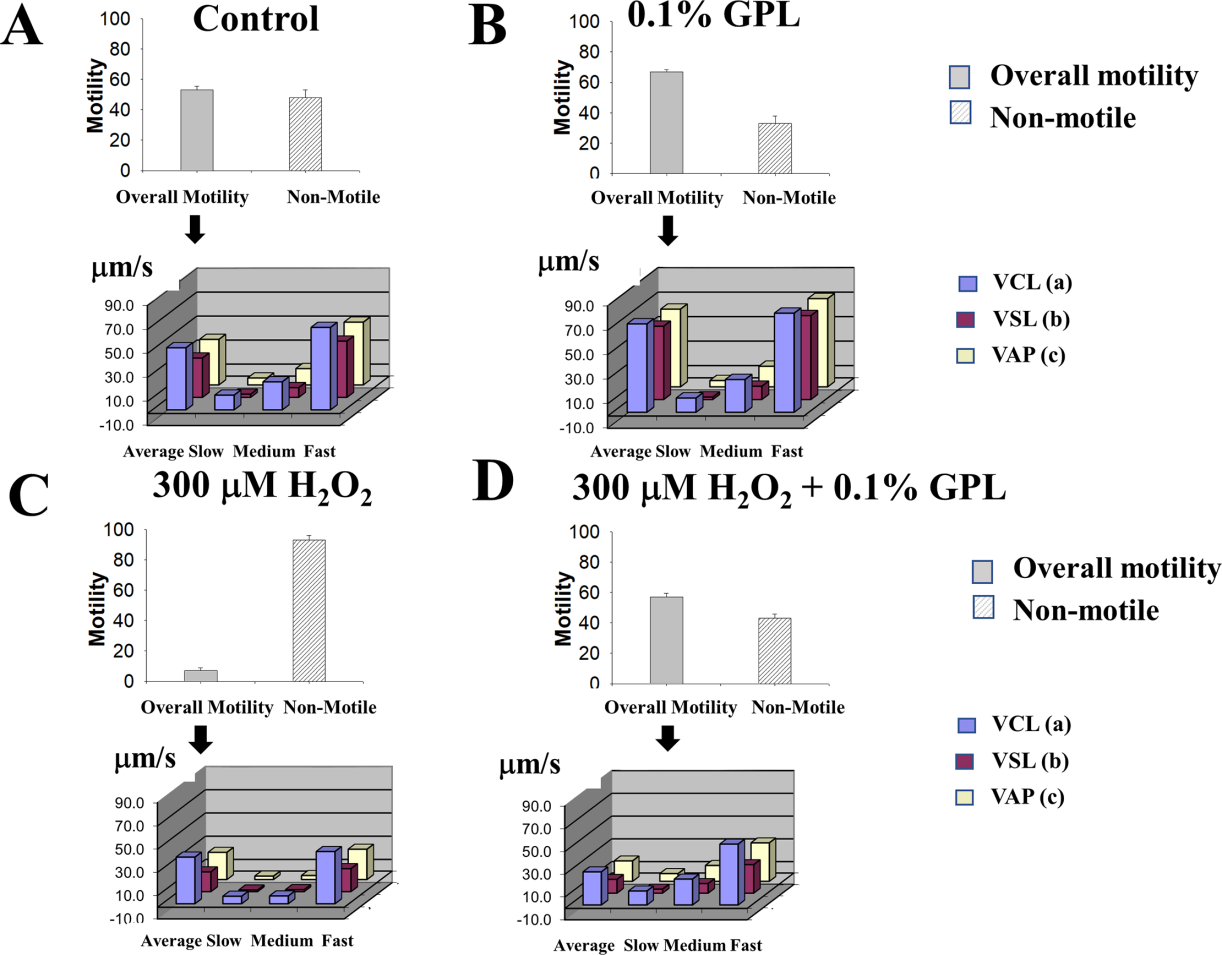
Analysis of motility of mature human sperm from adult males incubated under different experimental conditions (control, GPL, H_2_O_2_, GPL plus H_2_O_2_). For each experimental condition (listed on the top of each graph set) the percent overall motility (grey bar) and immotile sperm (dashed white bar) are shown. Below these plots are shown the curvilinear velocity (VCL), the straight line velocity (VSL) and the average path velocity (VAP) in μm/s, for each type of sperm motile pattern (average, slow, medium and fast motility)(n = 8 experiments). (A) control (B) 0.1% GPL (C) 300 μM H_2_O_2_ (D) 300 μM H_2_O_2_ plus 0.1% GPL.

In Fig 5, the average motility percentage and the distribution bar charts of sperm velocities in μm/s are presented for the WHO velocity variables obtained by CASA under various experimental conditions. In Fig 5A, the control sperm that are motile were found to be approximately 52±2.5% of total sperm, after an incubation period of 3 h in HAM F10 medium (n = 8). We analyzed the distribution of velocities for the motile fraction of sperm. Motile sperm, according to the WHO, can be fast, medium or slow progressive [79]. The plot of velocity versus the type of motility for the three sperm velocities (following WHO standards), automatically reported by CASA analysis, are shown for a control incubation. Each velocity is indicated next to the bars (VCL, VLS, and VAP, shown as blue, purple and yellow), and it contains the average velocity for the motility and its distribution as fast, medium or slow velocities (Fig 5A). The same analysis was performed for sperm incubated for 3–4 h in sonicated 0.1% GPL (Fig 5B). When ultra-sonicated 0.1% GPL were present during incubation, the overall motility fraction increased from 52±2.5 to 68±1.34%, and the non-motile sperm decreased from 48±5 to 32±5% (n = 8 experiments, p<0.001). In addition, there was also a statistically significant increase of the fastest motility produced by incubating sperm in sonicated 0.1% GPL (68% compared to 52%) (n = 8 experiments, p<0.001). The results indicate that incubation in 0.1% GPL is non-toxic for the sperm, as monitored by their motility. Moreover, this increase could be beneficial, as it increased significantly their fastest motility for all velocities examined (VAP, VCL and VLS). Sperm motility is one of the best indicators of semen quality [92]. We promoted oxidative stress by addition of 300 μM H_2_O_2_ for 3 h to the incubation medium and found that the motility monitored by CASA [79, 93] was significantly reduced (Fig 5C). Overall motility (grey) was reduced to 7±2% and immotile sperm (dashed white) increased to 93±2% of the sperm (n = 8,p<0.0001). All of the velocities of all types were dramatically reduced in agreement with previous reports [59, 94]. The average sperm velocities diminished dramatically to about 6 μm/s (n = 8, p<0.01), except for the fast velocities (30 μm/s). However, co-incubating the sperm with 300 μM H_2_O_2_ containing additionally 0.1% ultra-sonicated GPL, increased the overall motility from 7% to 58% and decreased the non-motile sperm from 93% to 42% (n = 8 experiments, p<0.01) (Fig 5D). Though all the velocities were increased for each type of motile sperm, the differences were more pronounced for slow and medium velocities (increases from 6 μm/s to 10 and 20 μm/s), whereas the fast velocities increased from 30 μm/s to 42 μm/s (see Fig 5D). These results suggest that ultrasonicated GPL can prevent the damage to sperm and their motility by agents that promote oxidative stress, such as H_2_O_2_. Thus, exposure to 0.1% GPL ameliorates the reduction of motility observed by exposure to H_2_O_2_, suggesting that the GPL mixture it is an effective agent to protect sperm against oxidative stress (one of the main causes of infertility).

**Fig 6:**
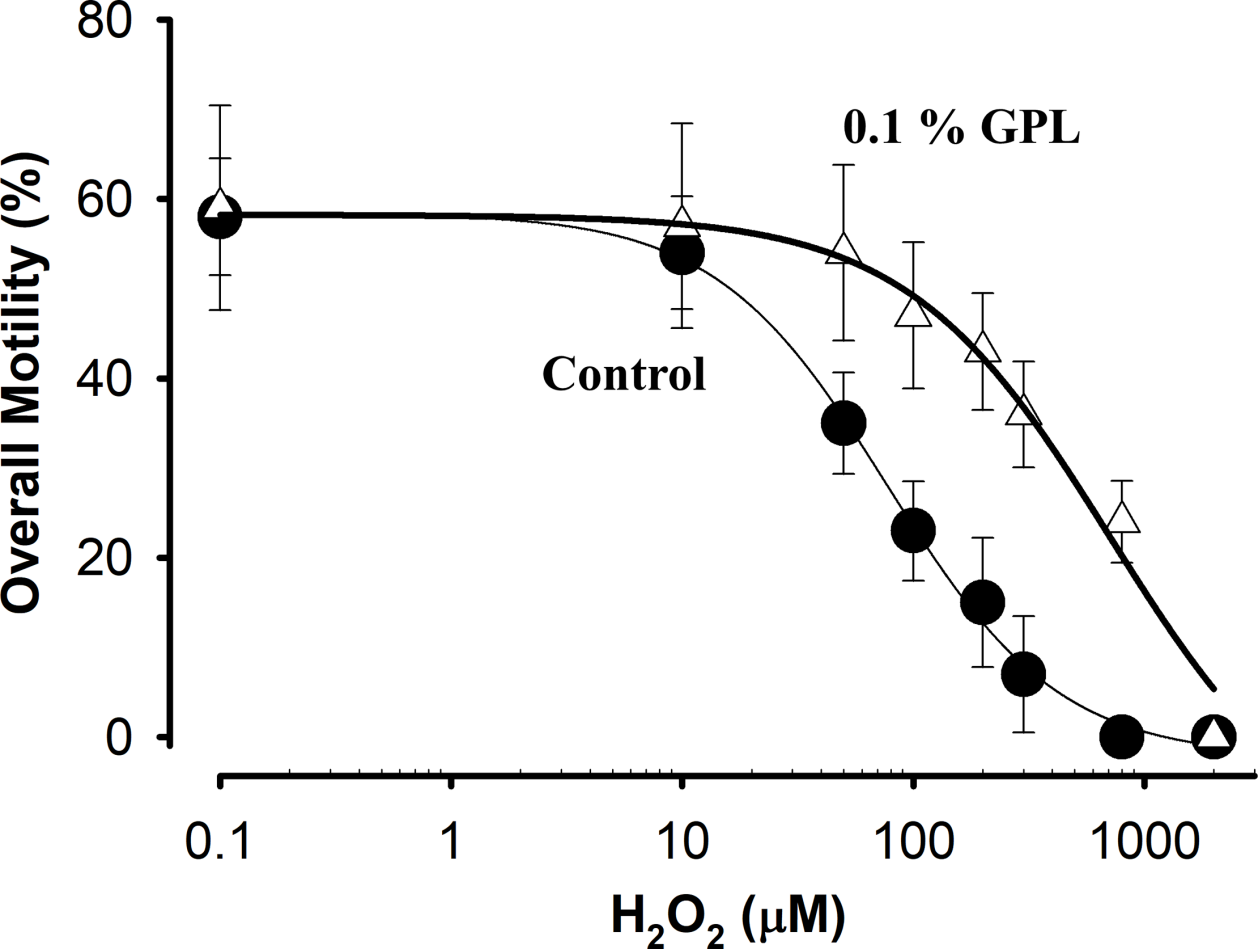
Dose response curves of overall motility versus hydrogen peroxide concentration. The data correspond to the average motility obtained after incubation in the presence of varying concentrations of H_2_O_2_ (solid circles) or in the presence of varying concentrations of H_2_O_2_ with 0.1% GPL added (open triangles) (n = 8 experiments). The solid lines represent the best fit of a Hill equation to the data.

We next examined the protective effect of ultra-sonicated 0.1% GPL on sperm motility when incubated together with different H_2_O_2_ concentrations. Dose-Response curves of sperm motility versus increasing H_2_O_2_ concentrations in control and in the presence of 0.1% GPL are shown in Fig 6. Overall motility was analyzed after an incubation period of 3 h. The effects of H_2_O_2_ concentrations on overall motility for the control (filled circles, without GPL) and for the incubation in 0.1% GPL (white triangles) are shown. The solid line represents the best fit of a Hill equation. The IC_50_ for overall motility changes from 80±15 to 700±20 μM when 0.1% GPL are added. The protective effect of GPL was observed at all concentrations of H_2_O_2_ tested. The results suggest a protective role of GPL against oxidative stress affecting sperm motility when they are exposed to different concentrations of H_2_O_2_.

#### Incubation of human sperm with a GPL mixture reduces the loss of mitochondrial membrane potential promoted by incubation with hydrogen peroxide

**Fig 7:**
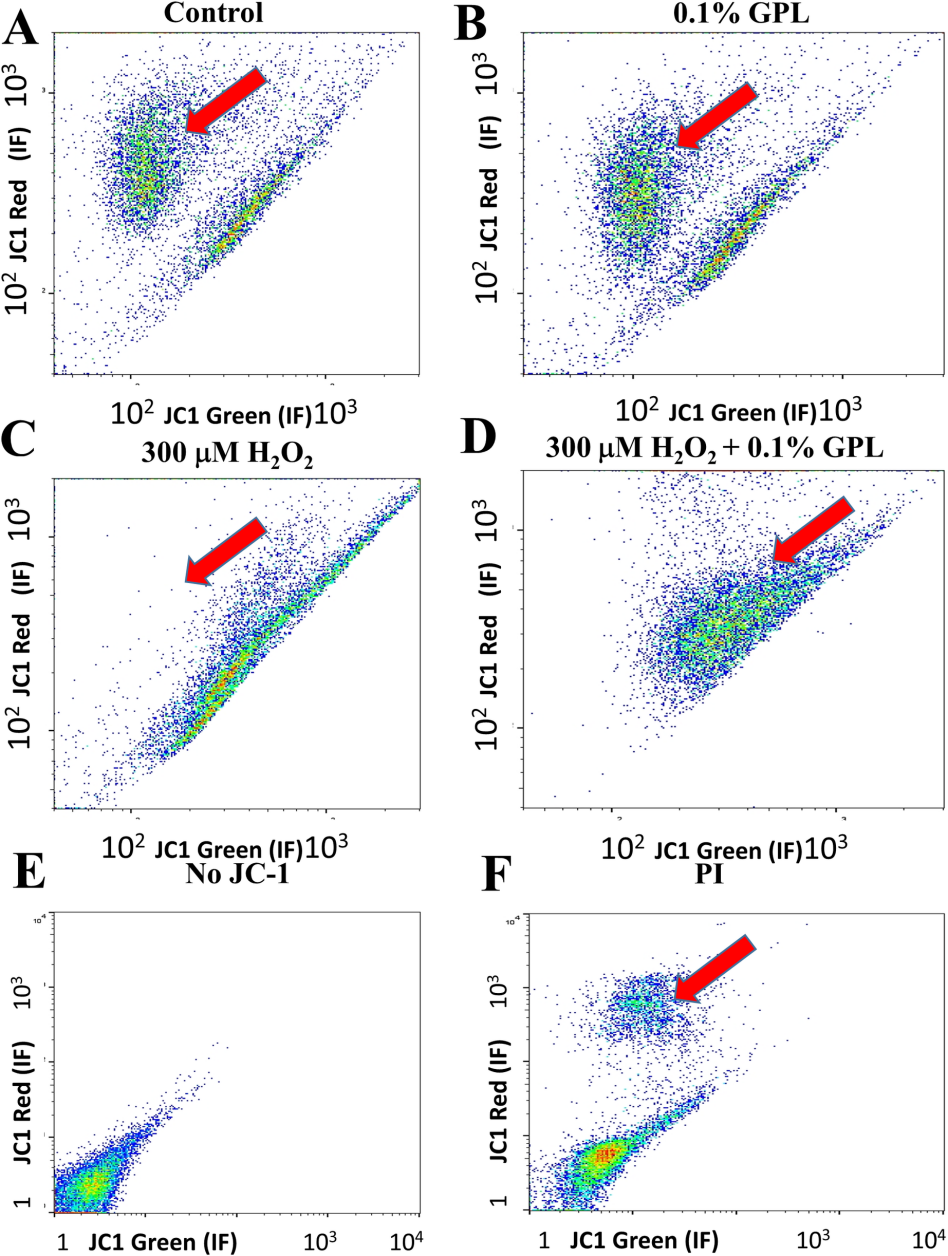
Dot plots from flow cytometry of sperm loaded with JC-1, a mitochondrial reporter, under different experimental conditions. (A) Results obtained after control incubation of sperm. (B) Incubation of sperm with 0.1% GPL. (C) Incubation of sperm with 300 μM H_2_O_2_. (D) Incubation of sperm with 300 μM H_2_O_2_ with the addition of 0.1% GPL. (E) Control obtained with unstained sperm (without JC-1). (F) Cells stained with JC-1 and exposed to the toxic molecule PI.

Since the viability and motility of sperm are related to the health of their mitochondria [95, 96], and because we have observed incorporation of GPL in regions of sperm where mitochondria are abundant, we sought to determine if these effects are correlated with mitochondrial function. Moreover, most of the ROS/RNS damage in sperm is linked to mitochondrial dysfunction. To examine if GPL can restore mitochondrial function in a population of sperm we performed flow cytometry of sperm loaded with JC-1, a fluorescent dye reporter of MIMP. Maintenance of MIMP is directly related to mitochondrial function [97]. Since JC-1 fluorescent ratio at 535/595 nm is directly related to MIMP, the red-to-green dye ratio of JC-1 was examined in a mixture of sperm incubated under different conditions: various control samples, 0.1% GPL, 300 μM H_2_O_2_, 300 μM H_2_O_2_ plus 0.1% GPL and exposure to the toxic agent propidium iodide (PI). JC-1 is a ratiometric dye, and the greater the ratio of red versus green intensities for the cells, the healthier they are; thus as the ratio red/green fluorescence increases, the more negative is the MIMP, and the more high-energy molecules produced. Fig 7 shows comparisons in the log-log scale of individual sperm red-to-green fluorescence for each experimental condition. The data are represented from blue, green, yellow to red dots indicating lower to higher densities/probabilities of the results obtained with sperm for each pair of fluorescence intensities for red and green emission. Higher ratios of red versus green emission indicate healthier spermatozoids, because the distribution reports collectively a more negative MIMP. Two types of data point clouds can be distinguished. One type of data cloud can be found on a diagonal following an almost direct proportional ratio of red-to-green fluorescence with a slope of approximately 1. These sperm, are likely to be damaged or at least not in a healthy condition (Fig 7A). The red line separates another cloud of data points with a higher red/green fluorescent ratio, indicating functional maintenance of the MIMP. These are mostly healthy sperm (see large red arrows). In Fig 7B the same experiment was performed with sperm were incubated with 0.1% GPL. Since the results are similar to the control in Fig 7A, GPL must be non-toxic to the sperm and may even increase slightly their viability. This is apparent because the cloud of healthy spermatozoids in the GPL-treated sample has the same or larger area than that observed in the control, suggesting that GPL treatment can improve the control viability. When sperm were incubated with a toxic oxidizing agent (300 μM H_2_O_2_), the cloud of healthy sperm almost disappears, and only the diagonal cloud corresponding to non-healthy sperm remains (Fig 7C). The addition of 0.1% GPL to the incubation along with 300 μM H_2_O_2_ promotes the appearance of healthy sperm (red arrow, Fig 7D). This plot is clearly different from that observed after incubation with 300 μM H_2_O_2_, suggesting a protective role of GPL against the loss of MIMP promoted by H_2_O_2_. The plot in Fig 7E corresponds to the experimental control without JC-1 and indicates that the low levels of fluorescence shown at the left-bottom of each panel represent nonspecific emission. To evaluate sperm cell viability and membrane integrity an intercalating fluorescent agent (PI) was applied during the incubation with the JC-1 dye. PI is also toxic to cells, promoting cell death [98]. Although most of the sperm died during the incubation, yielding a pattern similar to JC-1-unstained sperm, some sperm survived corresponding to the position of the cloud shown in Fig 7A and 7D (red arrow, Fig 7F).

**Fig 8:**
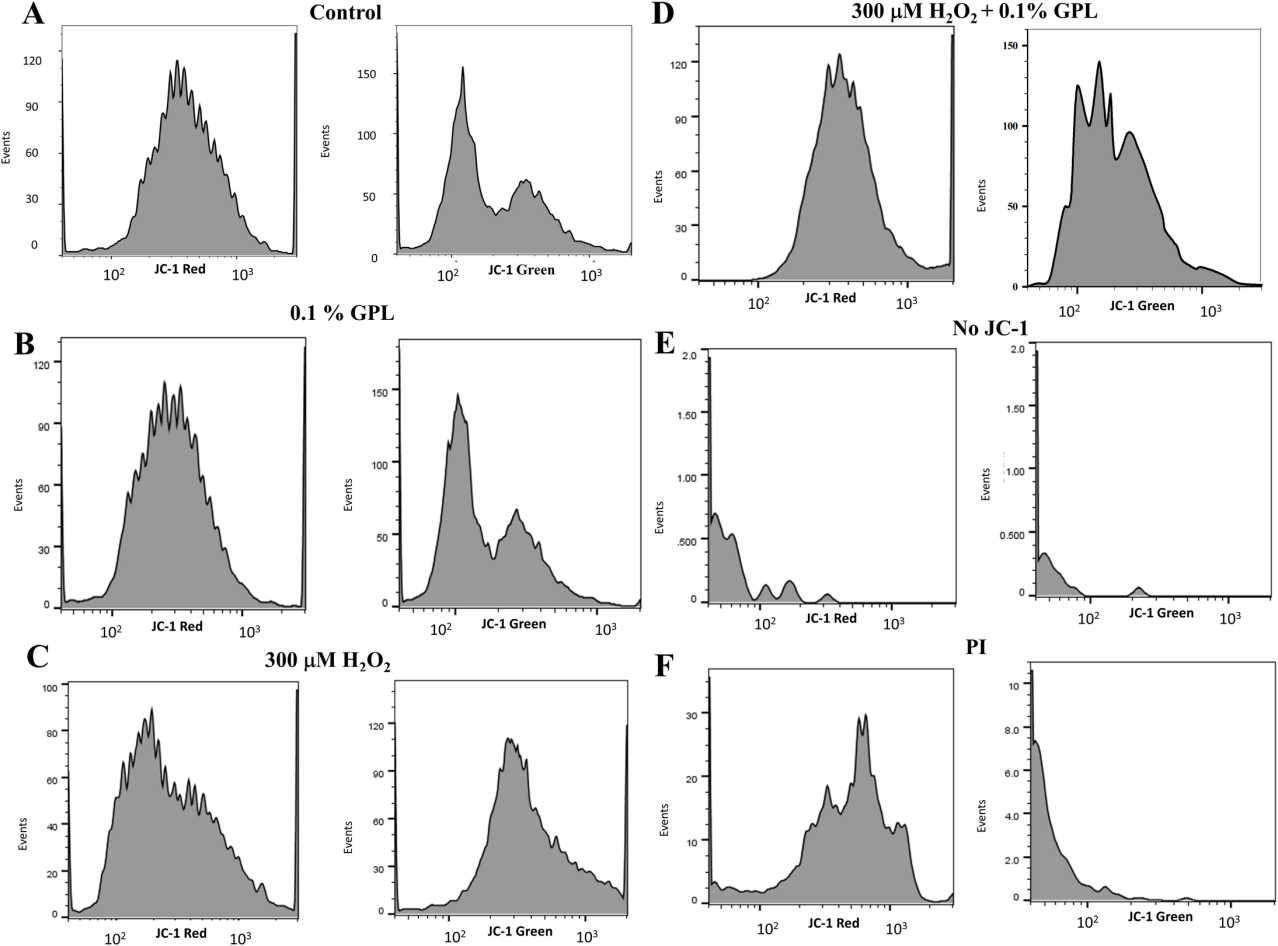
Histograms of red and green fluorescence intensity of sperm stained with JC-1. (A) Histograms of fluorescence of sperm red (left panels) and green (right panels) fluorescence intensities obtained after the control incubation (semi-log scale). (B) Fluorescence histograms of sperm obtained after incubation with 0.1% GPL. (C) Fluorescence histograms of sperm obtained after incubation in 300 μM H_2_O_2_. (D) Fluorescence histograms of sperm after exposure to 300 μM H_2_O_2_ in the presence of 0.1% GPL. (E) Fluorescence histograms of sperm cells without JC-1. (F) Fluorescence histograms of sperm in the presence of propidium iodide (PI).

Flow cytometry fluorescence histograms using JC-1 plot the number of events for red (left panels) and green (right panels) fluorescent intensities under different experimental conditions (Fig 8). The area under each curve represents the number of sperm found at different fluorescence intensities (semi-log plot). Fig 8A shows the control histograms. There is only one red fluorescence peak at an intensity of 400 arbitrary units (Fig 8A left panel). The range of events is distributed at fluorescence intensities between 100 and 2000 (log scale). There were two peaks in the green fluorescence histogram, one at 100 and another at 400 arbitrary units (Fig 8A right panel). The red-to-green ratio for the lower peak was approximately 4, suggesting that the highest peak at a lower green intensity corresponds to healthy sperm. When comparing the distribution of red versus green fluorescence in both plots, the green fluorescence peak that corresponds to the red fluorescence peak was lower than the other green fluorescence peak, and therefore this lower peak represents unhealthy sperm with lower MIMP. In Fig 8B the same results were obtained after incubation of the sperm with 0.1% GPL (red fluorescence peak ~ 350 arbitrary units, same values for green). This result indicates that the GPL does not promote damage to the sperm. In Fig 8C the sperm were incubated with 300 μM H_2_O_2_. The red fluorescent intensity was shifted towards lower values with a peak at about 130 arbitrary units, and the green fluorescent intensity became one peak centered at about 300 arbitrary units of fluorescence intensity. These results indicated that most of the sperm have a dramatically lower ratio of red/green fluorescence after treatment with H_2_O_2_, suggesting a dramatic loss of MIMP and an increase in unhealthy sperm. Fig 8D shows the effect of adding 0.1% GPL to the sperm in the presence of 300 μM H_2_O_2_. The peak for red fluorescence was shifted towards 250 fluorescence intensity units, whereas the histogram for green fluorescence revealed a bimodal plot similar to that observed in Fig 8A and 8B. The peak at a lower intensity was positioned at about 120 arbitrary units, whereas the peak at a higher intensity was positioned at about 300 arbitrary units. The reappearance of two peaks of green fluorescence suggested a protective effect of the GPL from the oxidative stress produced by 300 μM H_2_O_2_. In Fig 8E the control histograms for red and green fluorescence without the addition of JC-1 are shown. Thus, the fluorescence measured in Fig 7 and Fig 8A–8D were due to JC-1 and were not artifacts. In Fig 8F, the histograms are shown after incubation with PI. PI molecules can enter sperm that have lost membrane integrity and are also toxic to sperm. With PI treatment the red fluorescence peak was observed at approximately 700 arbitrary units, corresponding to surviving viable sperm. The number of events in the vertical axis in Fig 8F are quite low in comparison with Fig 8A–8D, and the green fluorescence is also low, mostly at intensities below 100, indicating the presence of non-viable sperm after exposure to PI. This control indicates that the results in Fig 8A–8D reflect measurements with viable sperm.

In summary, the incubation of human sperm with a GPL mixture reduced the loss of MIMP after incubation with hydrogen peroxide. Similar incubations were performed using Rhodamine 123 and JC-1, and the sperm were examined under a confocal microscope. The results confirmed the cytofluorographic studies.

**Fig 9:**
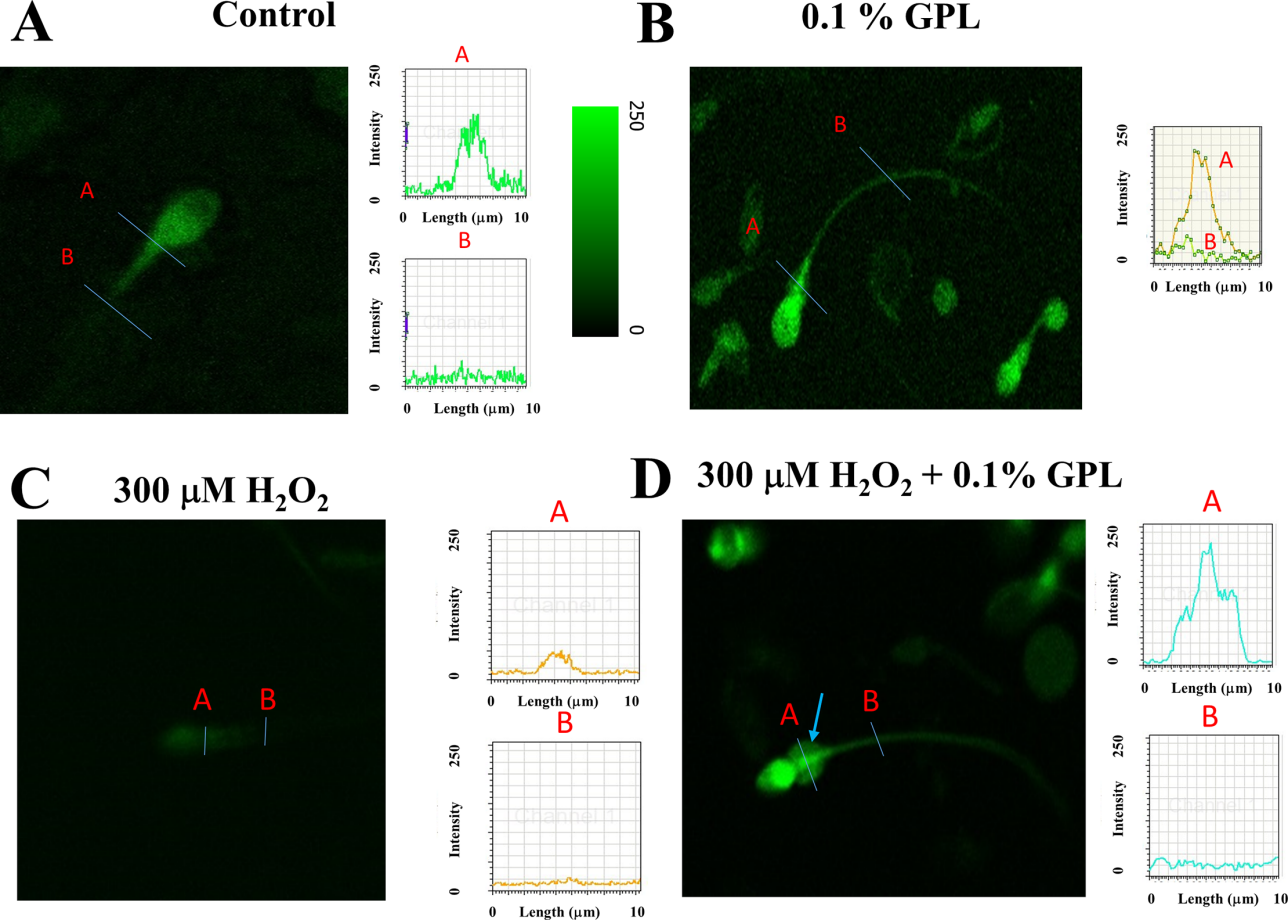
Confocal images of human sperm stained with Rhodamine 123 under different experimental conditions. Segment A has been inserted in a sperm sector enriched in mitochondria, whereas segment B has been placed in a sector devoid of mitochondria. Fluorescence intensity plots from segments A and B are shown to the right. (A) A typical spermatozoon stained with Rhodamine 123 under control conditions. (B) A sperm stained with Rhodamine 123 in medium containing 0.1% GPL. (C) A typical spermatozoon stained with Rhodamine 123 in medium containing 300 μM H_2_O_2_. (D) A sperm stained with Rhodamine 123 in 300 μM H_2_O_2_ with 0.1% GPL.

Flow cytometry has the advantage of examining thousands of cells under different experimental conditions, but it cannot evaluate the origin of the fluorescent signal. To better understand this, as well as the improvement of viability over time in sperm incubated with GPL we examined sperm under a confocal microscope after loading with dyes that can evaluate MIMP. In Fig 9, a typical sperm loaded with the Rhodamine 123 dye under different conditions for 3 h (control, 0.1% GPL, 300 μM H_2_O_2_ and 0.1% GPL plus 300 μM H_2_O_2_) is shown. Though in each panel we show just one sperm cell stained with the dye Rhodamine 123, we performed the analysis under each condition for 150 sperm randomly selected from different fields for each sperm sample. After incubation in control media, segments A and B of the sperm were rich in or devoid of mitochondria, respectively (Fig 9A). The fluorescence intensity for those sperm segments A and B are shown to the right with the same scale for the ordinate axis. The higher the fluorescent intensity observed in segment A, the healthier the mitochondria. The pseudo-color intensity scale is also shown. The peak of fluorescence intensity in segment A is between 150 and 200. Similar results were observed in 59±5% of the total number of randomly selected sperm analyzed under this experimental condition (n = 8 samples). Fig 9B shows a confocal image of sperm after incubation with 0.1% GPL and stained with Rhodamine 123. The neck of a sperm (segment A) has an intense fluorescence intensity (peak A, to the right), compared with other regions of the sperm (such as segment B shown in peak B, to the right). The fluorescence intensity in the dye-intense regions can reach values of approximately 200 arbitrary units (see graph to the right) [99–101]. Similar results were observed in 64±7% of the total number of randomly selected sperm analyzed under this experimental condition (n = 8 samples). Another interesting point is that when sperm were incubated with GPL, a cytoplasmic droplet appeared in many sperm cells. It has been suggested that such cytoplasmic droplets are indicative of stronger resistance to oxidative stress and other damaging events [102–104]. Sperm membrane channels and signaling processes localized in the sperm head are essential for the acrosomal reaction and successful fertilization [104, 105]. In Fig 9C the results obtained after incubation of sperm in 300 μM H_2_O_2_ are shown. Most of the sperm loaded with the Rhodamine 123 dye showed low fluorescence intensity for segments in the sperm neck (segment A). The maximum values observed were around 50 fluorescence arbitrary units. This reduction implies that exposure to 300 μM H_2_O_2_ results in oxidative stress to mitochondria. The loss of staining suggests that exposure to 300 μM H_2_O_2_ results in unhealthy sperm due to the loss of mitochondrial function. Similar results were observed in 84±8% of the total number of randomly selected sperm analyzed under this experimental condition (n = 8 samples). Co-incubation of sperm with GPL plus 300 μM H_2_O_2_ restores the fluorescence values of segment A to 200 arbitrary units. The peak of fluorescence for segment A is in a similar range to those found in Fig 9A and 9B, suggesting again a protective role of GPL for the mitochondrial oxidative stress produced by H_2_O_2_. Most of the sperm have cytoplasmic droplets (see light blue arrows). It has been hypothesized that the cytoplasmic droplet appears as a response to environmental stress [102–104]. Similar results were observed in 54±3% of the total number of randomly selected sperm analyzed under this experimental condition (n = 8 samples). The results under confocal microscopy obtained with Rhodamine 123, under the same conditions as described in Figs 7 and 8, are consistent with the results described previously. This suggests that GPL incorporate into the sperm membranes and partition into the mitochondria, possibly removing and substituting GPL for peroxidized lipids.

**Fig 10:**
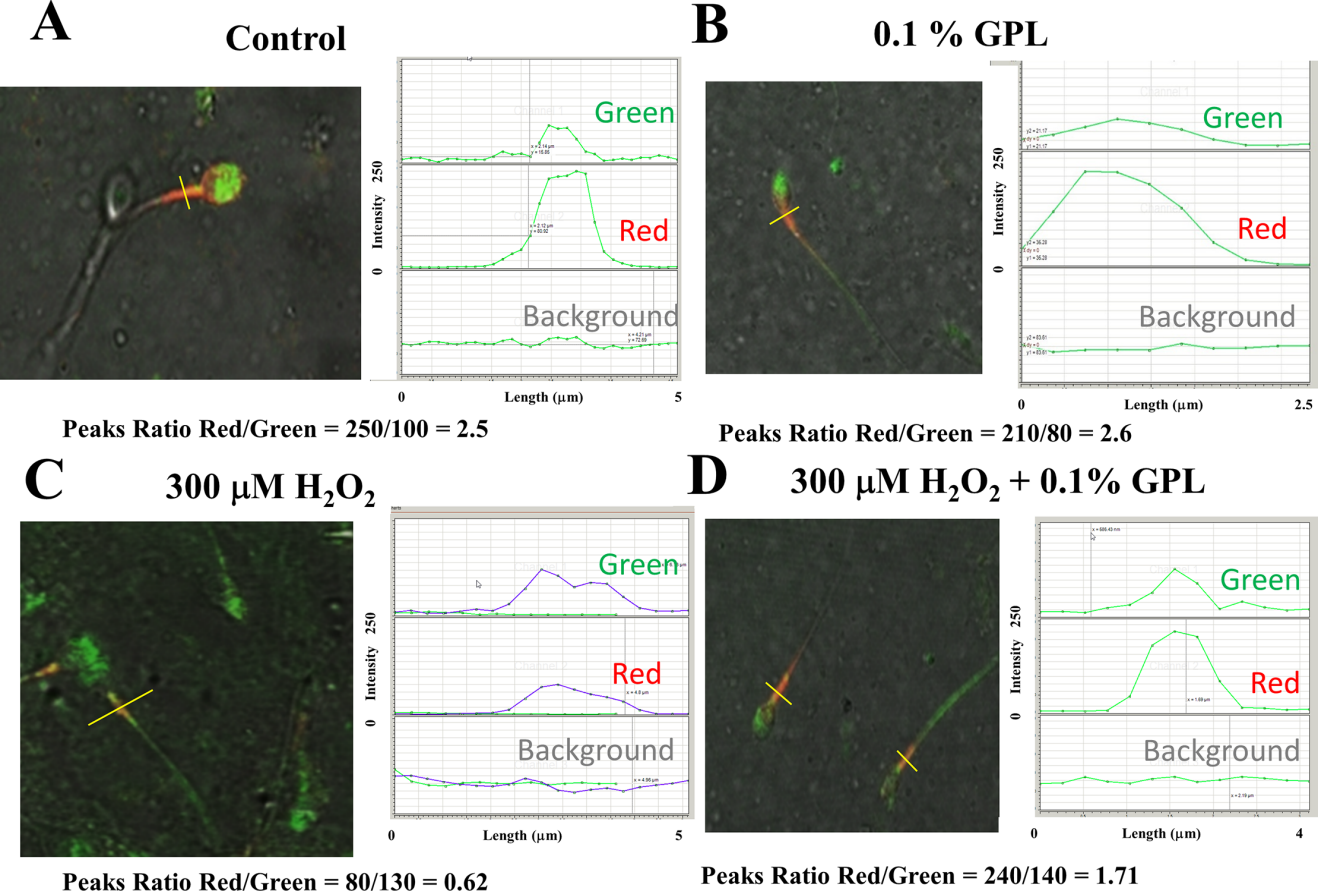
Confocal images of human sperm stained with JC-1 under different experimental conditions. (A) Live imaging of sperm incubated in control medium. (B) The same experiment as in A, but in the presence of 0.1% GPL. (C) Sperm incubated in 300 μM H_2_O_2_. (D) Sperm co-incubated with 300 μM H_2_O_2_ and 0.1% GPL.

To better assess the MIMP and fluorescence in different sperm segments, such as the neck/midpiece, we have used JC-1 instead of Rhodamine 123, along with acquiring images at low magnification to evaluate multiple sperm at the same time (Fig 10). This approach was applied to approximately 150 randomly selected sperm in different fields for each of the samples. A typical image of the stained sperm for each experimental condition is shown in each panel. Using JC-1, the red/green ratio of the peak signal at the midpiece was calculated to obtain an estimation of MIMP and thus the health of sperm (yellow lines). The plots to the right labeled as Green, Red, and Background, represent the levels of fluorescence intensity along the yellow segment through the spermatozoon, with the same scale for the ordinate axis. The red/green ratio was almost 2.5±0.1 for the control, indicating that mitochondria stained with JC-1 show negative MIMPs (<-100 mV) (n = 16, p<0.01) (Fig 10A) [99]. Similar results were observed in 55±3% of the total number of randomly selected sperm analyzed under this experimental condition (n = 16 samples). This same type of experiment was performed in the presence of 0.1% GPL, yielding a ratio of 2.6±0.15 (n = 14, p<0.01)(Fig 10B). The ratio red/green fluorescence was similar but showed a slight improvement to that seen in Fig 10A. Similar results were observed in 60±8% of the total number of randomly selected sperm analyzed under this experimental condition (n = 14 samples).The incubation in 300 μM H_2_O_2_ shows mitochondrial oxidative stress and loss of the MIMPs to less than -50 mV (Fig 10C). The ratio red/green falls to approximately 0.62±0.12 (n = 10, p<0.01). Similar results were observed in 89±5% of the total number of randomly selected sperm analyzed under this experimental condition (n = 8 samples).Fig 10D shows that the addition of 0.1% GPL to the incubation with 300 μM H_2_O_2_, restored, at least partially, the red/green ratio to 1.7±0.18 (n = 16, p<0.01). Similar results were observed in 56±4% of the total number of randomly selected sperm analyzed under this experimental condition (n = 16 samples).This result is consistent with a protective effect of GPL against mitochondrial oxidative stress [64–66]. Again, these experiments agree with the notion that the GPL have a protective effect against mitochondrial oxidative stress promoted by H_2_O_2_.

## Discussion

Membrane Lipid Replacement (MLR) has been used as a novel therapeutic approach to substitute damaged lipids in biological membranes [53, 62]. It has been shown to be useful to ameliorate the symptom severity in many chronic diseases, such as fibromyalgia, chronic fatigue syndrome, metabolic diseases, among others [53, 106–108]. It has been hypothesized that fresh, undamaged GPL can substitute for damaged GPL in biological membranes. Damage to GPL in biological membranes is an inevitable process that occurs naturally during cell aging or may be increased in pathological conditions [109–111]. Mitochondrial membranes are especially susceptible and relevant targets in these processes, as they are one of the main sources of ROS, which in turn is one of the leading causes of membrane lipid peroxidation [109]. It has been suggested by indirect evidence that MLR can replace damaged lipids, not only in the plasma membrane but also in mitochondrial membranes [54, 107, 108, 112]. However, there remains a lack of experimental evidence showing the incorporation of GPL into biological membranes (plasma and intracellular membranes). One goal of this report was to prepare GPL in a suitable way to create sub-μm-sized micelles that can be used to collect direct optical and functional experimental evidence of the incorporation of GPL into biological membranes through MLR. Once we established that, another aim using sperm as model cells was to test if MLR with GPL can diminish the functional damage produced by oxidative stress and reduce the impact of oxidizing agents on mitochondrial membranes. Our results suggest that incubation of sperm with sub-μm-sized micelles prepared with a GPL mixture can result in the incorporation of GPL into the SM. In addition, incubation with this mixture reduced the damage of oxidizing agents and increased the motility of sperm, while preventing the loss of MIMP produced by these agents.

### Physical evidence of GPL incorporation into sperm membranes

We created sub-μm-sized GPL micelles through ultrasonication and subsequent purification through size exclusion chromatography as described in methods and observed in Fig 1. We chose spermatozoa as model cells to directly test MLR and the incorporation of fresh GPL into their membranes. Sperm GPL are abundant with PUFAs that alter sperm function, and sperm can be monitored when their membranes are oxidized. In addition, they are individual cells that are easy to evaluate in terms of their viability and function, and they are motile, scanning all particles at the surface where they move. Sperm motility is also critically dependent on the constitution of SM lipids and on the health of their mitochondria [27, 31, 47, 91, 113, 114]. Thus the incorporation of GPL into sperm membranes was considered much easier to follow than other cell types where features of the cell morphology are not so distinct. The incubation of sperm with different concentrations of sub-μm-sized GPL micelles resulted in increases in the area of the head (see Fig 2). Previous MLR studies have shown that it is likely that MLR GPL are distributed into all cellular membranes, but the estimation of the incorporation of GPL from the sub-μm-sized micelles into SM was only detectable on the basis of membrane surface area in the head of the sperm. As the first SM to contact sub-μm-sized micelles are generally the plasma membrane of the head, it is likely that sub-μm-sized micelles deposit more GPL there as well.

The energy needed for lipid incorporation into membranes may also change with the degree of curvature of the membrane [115]. Thus, MLR GPL incorporation into SM may be heterogeneous in different membranes and even in the same membrane in different regions. To directly observe the incorporation of GPL from the sub-μm-sized micelles into SM we prepared a crosslinked GPL mixture with Rhodamine 123 using EDC water soluble ceramides [75, 78]. This procedure has been used before with liposomes to report lipid incorporation into mitochondrial membranes [72]. After these procedures, the GPL-linked Rhodamine 123 was still fluorescent and formed sub-μm-sized micelles as before (see Fig 3). Our experiments suggested that GPL from sub-μm-sized micelles incorporated into the SM. We next gathered functional evidence of GPL incorporation into SM by monitoring functional changes, such as changes in sperm motility.

### Functional evidence of GPL incorporation into sperm membranes

Sperm plasma membranes have restricted spaces to free lateral diffusion [116], and there are differences in lipid compositions and fluidities of the plasma membranes in sperm in the head and other regions [8, 117]. However, there is also evidence suggesting that most of the membranes in sperm and other cells that show structural asymmetries are in contact with intracellular membranes and exchange lipids through direct contact and transfer by non-vesicular transport or other pathways [2, 16, 19, 118, 119]. Thus, the incorporation of GPL into the plasma membrane will likely impact intracellular membranes in sperm organelles, such as the residual nuclear envelope (RNE) and mitochondria. These organelles play important roles in the motility of sperm [113, 114, 120–125]. Damage to membrane lipids in these organelles and in the plasma membrane has been reported to diminish sperm motility [8, 55, 91, 126]. Hence, we determined if there were observable changes in basal conditions after incubating sperm with sub-μm-sized micelles containing GPL. Fig 4 examines the result of such an experiment, where incorporating GPL from sub-μm-sized micelles at different concentrations caused different effects. We observed a dual effect on sperm motility by increasing the concentration of GPL. At high GPL concentrations (>3%), the size of micelles started to interfere with the measurements of the motility of sperm, leading to confusing results. Even though we separated micelles from sperm in all our measurements, a small residual fraction remained. This small fraction became significant at high GPL concentrations, especially at concentrations >3%, interfering with the estimations of sperm motility. At lower concentrations, the size of the micelles did not interfere with the CASA measurements. It was at these concentrations that we observed significant increases in sperm motility with the incubation of GPL in sub-μm-sized micelles, especially when oxidative stress was promoted in the sperm by the addition of H_2_O_2_.

The richest SM areas in phospholipid contents are usually near the post-acrosomal regions around the sperm neck and the RNE [127]. The RNE is a calcium storage organelle, and it has been implicated as an important source for calcium release in sperm [128]. This is especially interesting because of the possible continuity in SM and the involvement of the RNE in the process of signaling within sperm [12, 128]. The RNE is also enriched in sterols and phosphoinositides [129]. These are important signaling molecules that are involved in calcium homeostasis, and they are also critically involved in many essential sperm functions, such as motility, capacitation, acrosome reaction and fertilization [128, 130–132]. Phosphoinositides constitute 25% of the GPL mixture. This is important because it suggests that MLR can affect or contribute to calcium homeostasis. Moreover, there are several plasma membrane ion channels implicated in the regulation of calcium homeostasis modulated by the surrounding lipid environment [133–136]. For example, CatSper channels are critical for many sperm functions, and they are dramatically modulated by progesterone, which in turn is critically dependent on the membrane lipid constitution for its appropriate action [136–140]. These results suggest several possibilities related to the mechanism of action of MLR, as it suggests that MLR can indirectly affect the functioning of several membrane proteins like ion channels and also effect intracellular calcium homeostasis.

### Protective role of GPL against the loss of function in motility degraded by oxidative stress

One of the main mechanisms of damage to membrane lipids leading to cell dysfunction is lipid peroxidation [141]. PUFA are especially sensitive to lipid peroxidation [142]. Lipid peroxidation results in several mechanisms that can result in cell dysfunction and death, including the loss of fluidity and organization of the membranes and increased non-specific water permeability of the membrane bilayer [143].

Compared to other membranes, SM are particularly enriched in PUFA [6–9]. Because of this, SM are particularly susceptible to oxidative stress [4, 23]. In human sperm, there are multiple causes of oxidative stress [144], and such stress has been implicated as a major determinant of male infertility [40, 41, 145–148]. Considering this and the results discussed above, we decided to promote oxidative stress in sperm by incubating them with H_2_O_2_ and testing to see if the incubation with GPL sub-μm-sized micelles can reduce ROS damage [58, 149]. Our functional test was to measure the motility of human sperm under four different incubation conditions (control, with GPL sub-μm-sized micelles, with H_2_O_2_ and with H_2_O_2_ and plus GPL sub-μm-sized micelles) (Fig 5) and to obtain a dose-response curve of sperm motility against concentration of H_2_O_2_ with or without GPL sub-μm-sized GPL micelles (Fig 6). The fact that motility during co-incubation of H_2_O_2_ plus GPL sub-μm-sized GPL micelles, can restore sperm motility to values close to those obtained for the control, suggests that MLR with GPL can prevent the oxidative stress promoted by H_2_O_2_ in the SM. Furthermore, the protection against oxidative stress by H_2_O_2_ occurred at all doses. It has been shown that one of the targets and mechanism of damage of H_2_O_2_ exposure in human sperm are phospholipids [24, 26]. Sub-μm-sized GPL micelles made from fresh GPL mixtures are resistant to peroxidation [62]. These results are consistent with the findings in Figs 1–4, suggesting that lipids from sub-μm-sized GPL micelles incorporate into SM and protect these membranes. Moreover, they also suggest that MLR with GPL might be useful for treating those cases of infertility where oxidative stress is increased. As healthy mitochondria are a key to healthy fertile sperm [150], we next tested if the incubation with GPL sub-μm-sized GPL micelles could influence sperm mitochondrial function.

### Protective role of GPL against the loss of MIMP promoted by oxidative stress

Once the added sub-μm-sized GPL micelles contact sperm plasma membranes, they can transfer their constituents directly to SM. However, transfer to internal membranes may occur via different mechanisms, such as non-vesicular lipid exchange and membrane-membrane contact [5, 18, 118]. Alternatively, GPL incorporated into the SM may activate metabolic or signaling pathways (through ion channels, membrane receptors or second messengers) that could have indirect effects on internal membranes. Liposomes that fuse with the plasma membrane have been used to show translocation of lipids between the mitochondria and the plasma membrane [72]. The endoplasmic reticulum, with functional continuity to the plasma membrane, is a major provider of mitochondrial lipids [151, 152]. Mitochondria and glycolysis are the main sources of energy production needed for sperm motility. Mitochondria are also one of the main sources of ROS involved in sperm oxidative stress [96, 144, 153]. Excessive ROS production in the mitochondria impacts sperm on many cell levels, among them are damage to lipids in the plasma membrane and mitochondrial membranes, resulting in loss of MIMP [154, 155]. An important cellular target of oxidative stress is cardiolipin, a major constituent of the mitochondrial inner membrane [156]. A key precursor of cardiolipin is phosphatidylglycerol, a component of our GPL mixture [62]. Providing precursors to critical mitochondrial molecules may be an important element in MLR enhancement in sperm function. There is indirect evidence that MLR with GPL can be a useful supplement in many chronic diseases, possibly by replacing damaged lipids in mitochondria [53, 54, 64, 66, 107]. We then decided to test if MLR with GPL incorporated into SM has a functional continuity that can impact internal membranes, either through lipid transfer or through the activation of pathways by the SM.

With this in mind, we took advantage of the easy access to the membrane systems in human sperm as a cell model and asked whether the loss of MIMP promoted by oxidative stress could be prevented by incubation with our mixture of GPL. Our first approach was to measure MIMP in a large population of sperm using flow cytometry of sperm loaded with the dye JC-1 that reports MIMP. The results presented in Figs 7 and 8 suggest that the GPL mixtures can prevent the loss of MIMP produced by H_2_O_2_, and it further suggests that the GPL reaches the mitochondria, replacing the damaged lipids with undamaged ones, restoring MIMP and mitochondrial function. We also tested this hypothesis in Figs 9 and 10, observing with a confocal microscope staining in mitochondria after incubating with dyes that report MIMP (Rhodamine 123 and JC-1).

We also found frequently cytoplasmic droplets in sperm incubated with GPL sub-μm-sized GPL micelles. In addition to suggesting that this could be evidence of GPL incorporation into the SM, it also suggests that GPL incorporation into internal membranes makes sperm more resistant to stress conditions. It has been suggested that the droplets are indicative of sperm oxidative resistance [102–104]. The same type of experiments using Rhodamine 123 were performed with the JC-1 dye with similar results indicating the type of dye used in the experiment did not affect the results. The ratio between incubation of sperm in control and the GPL mixture was similar, or even slightly better for the incubation with the GPL mixture. However, the ratio dropped to 0.62 when H_2_O_2_ was added, but the addition of the GPL mixture to the incubation with H_2_O_2_ restored the normal ratios. These results are consistent with the sperm motility and flow cytometry data and support the notion that MLR with GPL was incorporated into the mitochondrial membranes and prevented the loss of MIMP.

## Conclusions

Sub-μm-sized GPL micelles, like liposomes, can fuse with biological membranes and deliver phospholipids that can replace damaged membrane phospholipids in order to restore membrane function. Once in the plasma membrane, these replacement phospholipids can partition or be transferred to other membranes in a cell and restore function. Since this approach does not interfere with other treatment strategies, it can also be used to supplement other pharmacological approaches without the problem of interference or counter-indications that can occur with the use of different drugs.

This type of replacement approach is particularly useful for the treatment of excess oxidative stress. Oxidative stress is related to mitochondrial activity, and we have shown here that MLR with GPL can restore the mitochondrial function when it is damaged by oxidative stress.

Our results have shown that: (a) we can substitute the damaged GPL with a mixture of exogenous GPL in a combination that resembles the plasma membrane composition (b) we also show that this approach is valuable under basal conditions, but also when there is an increment of lipid peroxidation promoted by H_2_O_2_; and (c) we demonstrate that the plasma membrane and organelles in the sperm have functional continuity, because our treatment can modify mitochondrial membranes and change mitochondrial membrane potential.

Finally, our results suggest that MLR could be a relevant approach for the treatment of infertility in males when it is linked to increased oxidative stress.

## Supporting information

S1 Fig Elements found through X-Ray fluorescence. (A) Precipitated nano-micelles and spots picked for X-Ray fluorescence analysis (*up*) and number of counts *versus* energy plot (*down*). (B) Same as in (A) for a control surface.
(TIF)
