# Supplementary figures and images for: Incubation of human sperm with micelles made from glycerophospholipid mixtures increases sperm motility and resistance to oxidative stress
Source: PLoS One. 2018 Jun 1;13(6):e0197897. doi: 10.1371/journal.pone.0197897 (PMC5984032; doi:10.1371/journal.pone.0197897)

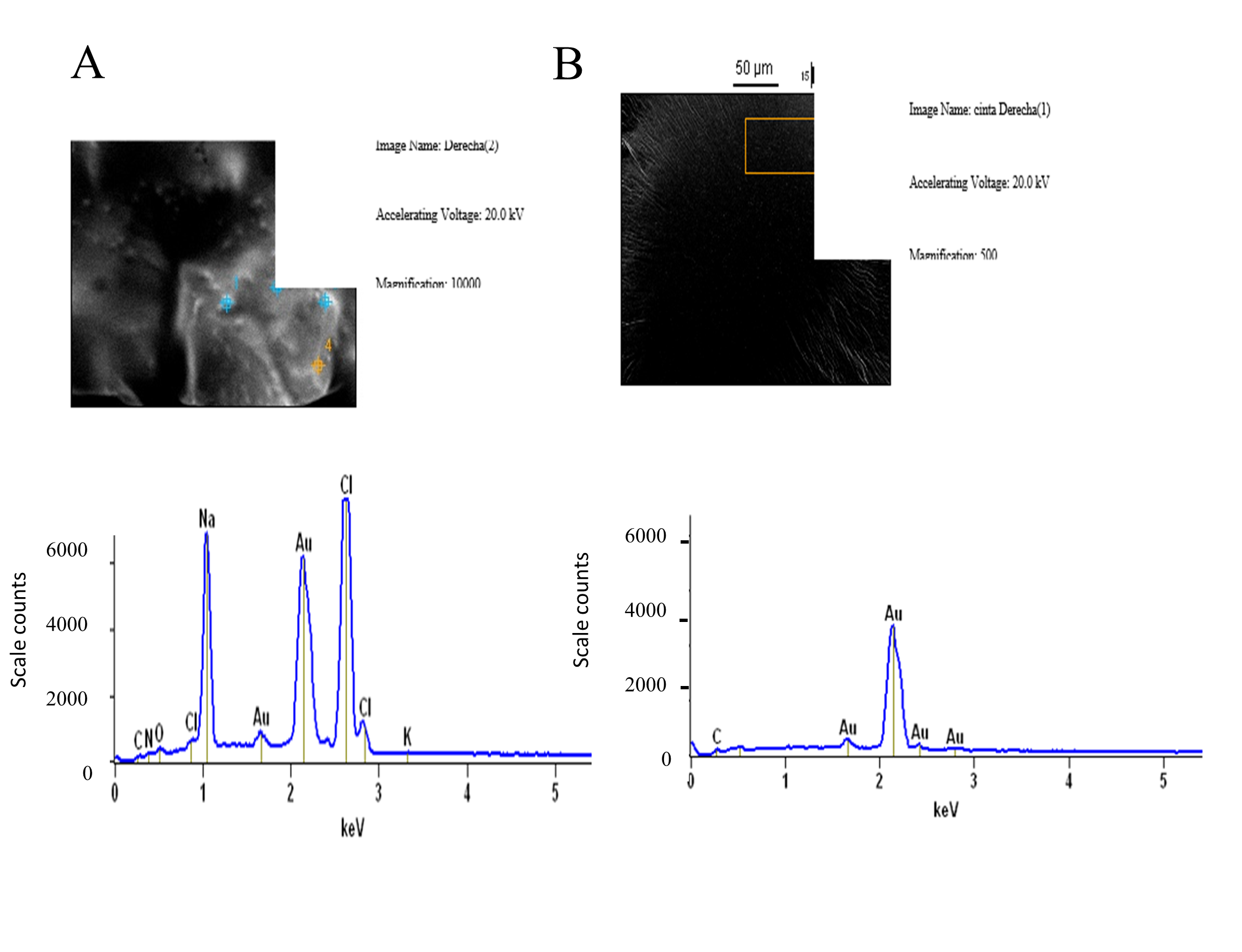

Supplement: S1 Fig — (A) Precipitated nano-micelles and spots picked for X-Ray fluorescence analysis (up) and number of counts versus energy plot (down). (B) Same as in (A) for a control surface. (TIF) [file pone.0197897.s001.tif]
